# Platelet–Leucocyte Aggregates as Novel Biomarkers in Cardiovascular Diseases

**DOI:** 10.3390/biology11020224

**Published:** 2022-01-30

**Authors:** Kinga Pluta, Kinga Porębska, Tomasz Urbanowicz, Aleksandra Gąsecka, Anna Olasińska-Wiśniewska, Radosław Targoński, Aleksandra Krasińska, Krzysztof J. Filipiak, Marek Jemielity, Zbigniew Krasiński

**Affiliations:** 1 1st Chair and Department of Cardiology, Medical University of Warsaw, Banacha 1a, 02-097 Warsaw, Poland; plutakinga.01@gmail.com (K.P.); ki.porebska@gmail.com (K.P.); 2 Department of Cardiac Surgery and Transplantology, Poznan University of Medical Sciences, 61-701 Poznan, Poland; tomasz.urbanowicz@skpp.edu.pl (T.U.); anna.olasinska@poczta.onet.pl (A.O.-W.); mjemielity@poczta.onet.pl (M.J.); 3 1st Department of Cardiology, Medical University of Gdansk, 80-210 Gdansk, Poland; rtargonski@gmail.com; 4 Department of Ophtalmology, Poznan University of Medical Sciences, 61-701 Poznan, Poland; alex.krasinska@gmail.com; 5 Department of Clinical Sciences, Maria Sklodowska-Curie Medical Academy in Warsaw, 00-136 Warsaw, Poland; krzysztof.filipiak@uczelniamedyczna.com.pl; 6 Department of Vascular and Endovascular Surgery, Angiology and Phlebology, Poznan University of Medical Sciences, 61-701 Poznan, Poland; zbigniew.krasinski@skpp.edu.pl

**Keywords:** aggregates, biomarker, cardiovascular disease, flow cytometry, leucocyte, platelets, platelet–leucocyte aggregates

## Abstract

**Simple Summary:**

Cardiovascular diseases are the most common cause of death worldwide. Hence, novel biomarkers are urgently needed to improve diagnosis and treatment. Platelet–leucocyte aggregates are conglomerates of platelets and leucocytes and are widely investigated as biomarkers in cardiovascular diseases. Platelet–leucocytes aggregates are present in health, but increase in patients with cardiovascular risk factors and acute or stable coronary syndromes, making them a potential diagnostic marker. Moreover, platelet–leucocyte aggregates predict outcomes after surgery or percutaneous treatment and could be used to monitor antiplatelet therapy. Emerging data about the participation of platelet–leucocyte aggregates in cardiovascular diseases pathogenesis make them an attractive target for novel therapies. Furthermore, simple detection with conventional flow cytometry provides accurate and reproducible results, although requires specific sample handling. The main task for the future is to determine the standardized protocol to measure blood concentrations of platelet–leucocyte aggregates and subsequently establish their normal range in health and disease.

**Abstract:**

Platelet–leucocyte aggregates (PLA) are a formation of leucocytes and platelets bound by specific receptors. They arise in the condition of sheer stress, thrombosis, immune reaction, vessel injury, and the activation of leukocytes or platelets. PLA participate in cardiovascular diseases (CVD). Increased levels of PLA were revealed in acute and chronic coronary syndromes, carotid stenosis cardiovascular risk factors. Due to accessible, available, replicable, quick, and low-cost quantifying using flow cytometry, PLA constitute an ideal biomarker for clinical practice. PLA are promising in early diagnosing and estimating prognosis in patients with acute or chronic coronary syndromes treated by percutaneous coronary intervention (PCI) and coronary artery bypass grafting (CABG). PLA were also a reliable marker of platelet activity for monitoring antiplatelet therapy. PLA consist also targets potential therapies in CVD. All of the above potential clinical applications require further studies to validate methods of assay and proof clinical benefits.

## 1. Introduction

Cardiovascular diseases (CVD) are the leading cause of death worldwide, responsible for over 17 million deaths in 2019 [1], which is the approximate number of inhabitants of the Netherlands [2]. Despite the recent development in the fields of prevention and treatment, CVD mortality increased globally from 27.9% in 2000 to 32.2% in 2019 [1]. Hence, novel biomarkers are urgently needed to predict CVD, improve early diagnostics, and monitor therapy effectiveness.

The formation of platelet–leucocyte aggregates (PLA) is one of the most crucial interactions between platelets and leucocytes [3]. PLA are defined as heterotypic combinations of at least one platelet with one leucocyte [4]. PLA are formed during immune or thrombotic reactions [5]. Hence, PLA have been investigated as potential biomarkers in many pathologies, including chronic obstructive pulmonary disease [6], antineutrophil cytoplasmic antibody (ANCA)-associated vasculitis [7], consumptive coagulopathy in xenotransplantation [8], cutaneous Arthus reactions [9], and infections [10]-among others COVID-19 [11]-and sepsis [12]. 

PLA have also been widely investigated in CVD. Levels of PLA, including monocytes or neutrophils, were higher in symptomatic coronary artery disease (CAD) than in healthy people [13]. PLA containing monocytes were increased in acute coronary syndromes (ACS) versus healthy subjects [14]. Similarly, levels of platelet-monocyte aggregates were higher in patients with non-ST elevation (NSTE-ACS), unstable angina (UA), acute myocardial infarction (AMI) or chronic coronary syndromes (CCS) compared with a group with non-cardiac chest pain [15,16]. Moreover, increased percentage of PLA-containing monocytes was associated with elevated risk for future cardiovascular events in population with NSTE-ACS or in dialyzed patients [15,17]. Increased levels of PLA were also observed in patients with carotid stenosis [18], peripheral artery disease (in which it was significantly higher in critical limb ischemia) [19,20], and a high risk of thromboembolic events [21,22]. Data regarding PLA in patients with abdominal aortic aneurysm is mixed [19,23].

There are variable methods for the assessment of PLA. Conventional flow cytometry (CFC) is the best approach in clinical conditions because of its wide availability and its fast and accurate measurement. The main disadvantages of the cytometry-based methods are ex vivo observation, lack of evaluation of PLA placement in tissue, and no direct tracing of intracellular interactions. On the other hand, imaging flow cytometry (IFC) or intravital microscopy avoid the limitations of CFC, but these techniques are more complex than CFC [24].

Here, we present a comprehensive analysis of PLA as novel biomarkers in CVD by discussing the characteristics of platelet–leucocyte aggregates, and their clinical applications, including the impact of cardiovascular risk factors and CVD on PLA, the predictive value of PLA in patients after percutaneous coronary intervention (PCI) or coronary artery bypass grafting (CABG), and changes in PLA concentration during antiplatelet therapy. Finally, we discuss the methods of PLA measurement, including flow cytometry and microscopy. 

## 2. Characteristics of Platelet–Leucocyte Aggregates

**Figure 1:**
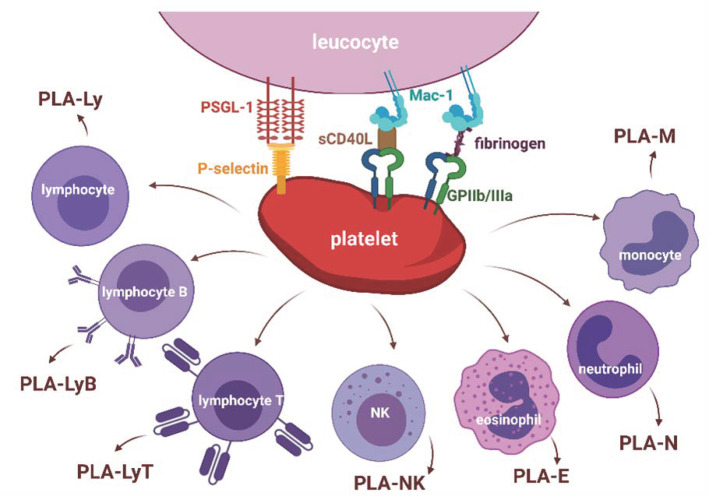
Molecules tethering platelets with leucocytes in platelet–leucocyte aggregates and subsets of platelet–leucocyte aggregates. Abbreviations: GP IIb/IIIa—glycoprotein IIb/IIIa; Mac-1—complement receptor 3; PLA-E—platelet-eosinophil aggregates; PLA-Ly—platelet–lymphocyte aggregates; PLA-LyB—platelet–lymphocyte B aggregates; PLA-LyT—platelet–lymphocyte T aggregates; PLA-M—platelet-monocyte aggregates; PLA-N—platelet–neutrophil aggregates; PLA-NK—platelet–natural killer cells aggregates; PSGL-1—P-selectin glycoprotein ligand; sCD40L—soluble ligand of cluster of differentiation 40.

PLA include various subtypes of leucocytes: granulocytes (PLA-G), neutrophils (PLA-N), eosinophils (PLA-E), monocytes (PLA-M), and lymphocytes (PLA-Ly)—B (PLA-LyB), T (PLA-LyT), T helper (PLA-LyTh), T cytotoxic (PLA-LyTc) as well as natural killer cells (PLA-NK) [4,25,26,27,28]. The subsets of PLA with binding molecules are shown in Figure 1.

Monocytes comprise three functionally distinct subtypes, which may contribute differently to CVD. Classical monocytes (M1) expose, on their surfaces, a high density of clusters of differentiation 14 (CD14), but do not present CD16. Intermediate monocytes (M2) also have a significant expression of CD14, but also expose CD16. Nonclassical monocytes (M3) expose CD16 as their primary markers, but are also positive for CD14 [29]. Intermediate monocytes (M2) are distinguished by a tremendous potential for reactive oxygen species formation. They also have high expression of CD74, a molecule essential for atherogenesis, and C–C chemokine receptor type 5 (CCR5), which is engaged in adhesion to endothelium and migration into atheroma [30,31]. Monocyte subsets could be distinguished even better by applying more surface markers including CD36, CCR2, human leukocyte antigen DR isotype (HLA-DR), CD11c [32] or transcriptomics [33]. However, there is great heterogeneity within the proposed subsets as well [33]. Nevertheless, the classification based on the expression of CD14 and CD16 has been so far widely used, including studies on PLA [20,21,23,34]. Classical as well as non-classical and intermediate monocytes could be tethered with platelets to create PLA [34]. Interestingly, platelets could also tether apoptotic leucocytes. In healthy people, apoptotic leucocytes were more frequently bound to platelets than present as free cells in blood [35]. 

The mean size of PLA is about 9 micrometres (µm) [36]. Following platelet activation, large conjugates were observed, consisting of many platelets in the central part and leucocytes, especially granulocytes and monocytes on their perimeters [4,37]. PLA could occur as free complexes or bound to vessels walls [38] and were detected in animals [39,40] as well as humans [41,42,43,44,45]. They were revealed in health [37,38] and many pathologies, including CVD [13,14,15,16,17,18].

In healthy populations, platelets form aggregates mainly with monocytes (4.1–7.2%), followed by neutrophils (3.7–5.7%) and lymphocytes (2.4–5.2%) [4,35,46], but the referential ranges are not known because of a lack of a validated protocol for investigating the concentrations of PLA. PLA were detected in the paediatric population [47] as well as in adults [48]. In patients with chronic coronary syndromes (CCS), there were no changes in PLA level dependent on age [48]. On the other hand, in the population with CVD, women revealed a higher concentration of aggregates [48,49], but this difference was not observed in healthy people [49,50]. In healthy women, PLA levels changed collaterally with estrogenic profile during the menstrual cycle. The highest percentage of PLA-M and PLA-G were at ovulation (14th day) in comparison to the 1st, 7th, and 21st days. Interestingly, the ability to create PLA by stimulation with thrombin receptor agonist peptide (TRAP) was decreased on the 14th day of the cycle, but there was no significant difference on the 7th and 21st days [51].

Activation of platelets or leucocytes led to PLA formation [50]. Biochemic substances associated with thrombosis, such as adenosine diphosphate (ADP), collagen, platelet-activating factor (PAF), and thrombin enhanced PLA level versus lack of stimulation [5,36,50]. Similarly, proinflammatory N-formyl-methionyl-leucyl-phenylalanine (fMLP), serotonin, and stromal cell-derived factor-1α (CXCL12) increased PLA formation versus lack of stimulation, but this effect was lower than induced by prothrombotic agents. Lipopolysaccharide (LPS) or interleukin 1β (IL1β), mediators of inflammation, boosted PLA creation only at low concentrations [5]. Stimulation of PLA formation with LPS was extended during elevation temperature for samples incubation from 37 to 38 °C. Further increase in the temperature to 39 °C decreased the adhesion of leucocytes to platelets, compared with 37 °C [52]. Other factors, including decreased pH (6.5–7.0) [53], shear stress [54], extracorporeal circulation [36], and vessels damage, also stimulated PLA formation [55].

On the contrary, low platelet-to-leucocyte ratio [56], nitride oxide (NO) [56,57], and C-type natriuretic peptide (CNP) [57] or methylation of platelet–endothelial aggregation receptor-1 (PEAR-1) [58] decreased PLA formation. Having a higher-than-physiological concentration of leucocytes inhibited PLA formation after stimulation with proteinase-activated receptor (PAR) agonist (receptor for thrombin on platelets). Interestingly, the NO was the main inhibitor of PLA creation [56]. NO donors: S-nitroso-glutathione [56] or sodium nitroprusside reduced thrombin-stimulated PLA formation [57]. CNP is released from endothelium and protects against injury during ischemia in the myocardium. Adding CNP to blood samples collected from healthy people decreased PLA formation stimulated by TRAP, compared with lack of CNP [57]. PEAR-1 is present among others on platelets and answers for intercellular interactions. The specific pattern of PEAR-1 methylation was negatively associated with PLA-M [58].

Surficial receptors participate in the adhesion of platelets to leucocytes [59,60]. Platelets, during activation, release granules containing P-selectin and are exposed on the surface P-selectin. Then P-selectin is tethered by a P-selectin glycoprotein ligand (PSGL-1) on leucocytes, leading to PLA formation [59]. On the other hand, PLA were also observed in mice without P-selectin, suggesting another mechanism of PLA creation [61]. Complement receptor 3 (CD11b/CD18, Mac-1) on leucocytes with participating soluble CD40 ligand or fibrinogen as the conduit was tethered with glycoprotein IIb/IIIa (GP IIb/IIIa) on platelets, creating an alternative means of PLA formation [60]. 

The release of inflammatory and thrombotic agents, such as free radicals, extracellular vesicles (EVs), and metalloproteinase (MP) and neutrophil extracellular traps (NETs), takes place during PLA formation. The creation of PLA in stimulation with PAR agonists at a leucocyte concentration similar to that in inflammation was associated with deriving free radicals. After adding a PAR agonist, PLA creation was observed with concomitant release of EVs [56], which are mediators of thrombosis and inflammation [62]. Furthermore, PLA contain on their surface MP 1-3,9. After stimulation with thrombin, PLA had more activated MP, and activated MP had greater activity than without stimulation. Moreover, active MP 1-3,9 caused PLA formation in stimulation with a low concentration of thrombin [56]. Activated platelets induce the release of NETs from neutrophils, which was demonstrated for various platelet agonists ( thrombin, TRAP-6, LPS) and conditions. Concurrent rise in PLA-N is usually reported and direct interactions between platelets and leukocytes may at least partly mediate this effect [63,64,65,66,67]. NETs in return generate thrombin potentiating platelet activation and provide a scaffold for an emerging thrombus [68].

## 3. Clinical Applications

**Figure 2:**
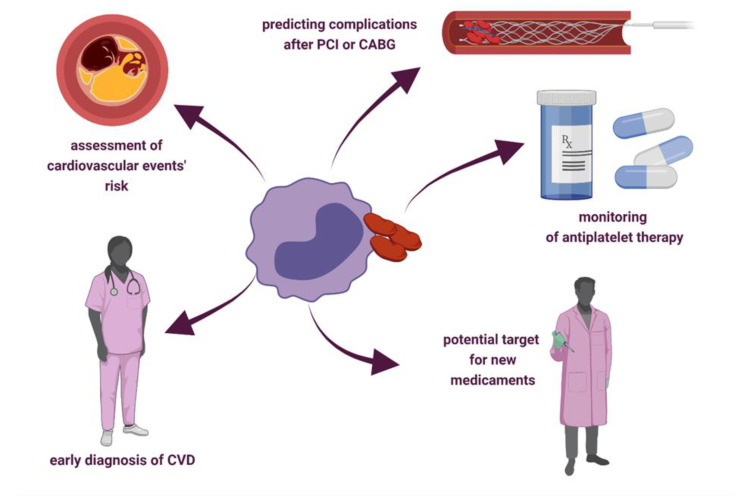
Potential clinical applications of platelet–leukocyte aggregates. Abbreviations: CABG—coronary artery bypass grafting; CVD—cardiovascular diseases; PCI—percutaneous coronary intervention.

PLA dynamics, diversity, and role were studied in several cardiovascular conditions, such as ACS, CCS, peripheral artery disease, heart failure, carotid artery stenosis, and cerebral ischemia [19,69]. The following paragraphs describe the relationship between PLA and cardiovascular risk factors, the potential of PLA as a biomarker to diagnose coronary syndromes, predict outcomes after revascularization, and monitor antiplatelet therapy. The potential clinical applications of PLA are shown in Figure 2.

In patients with CVD, the percentage of PLA-M, PLA-G, and PLA-Ly were increased compared with the group without CVD. In the population with CVD, PLA-M correlated with the number of atherosclerotic plaques and the mean intima-media thickness of the carotid artery. After 2 to 3 years of observation, people with CVD had decreased the percentage of PLA-M compared with their initial status, but still, PLA-M levels were higher than in a population without CVD at baseline. The percentage of PLA-M were similar in patients with CVD after 10 years of follow-up and without CVD at baseline [70].

### 3.1. Risk Factors of Cardiovascular Diseases

In a prospective cohort study including 155,722 participants from 21 countries followed-up for 9.5 years, 12 modifiable risk factors responsible for CVD development and mortality were shown: smoking, overusing alcohol, unhealthy diet, increased non -high-density lipoproteins (non-HDL), an increased waist–hip ratio (WHR), too infrequent physical activity, diabetes, hypertension, symptomatic depression, air pollution, decreased grip force, and lower education level [71]. 

In some of the above states, for example, in smokers [41,42], hypertension [43], symptoms of depression [44], or long-time exposure to biomass smoke, the concentrations of PLA were increased compared with the population without these states [45]. 

PLA-LyT, PLA-LyTh, PLA-LyTc, and PLA-E were increased in metabolic syndrome compared with no metabolic syndrome. In addition, PLA-LyTh positively correlated with plasma glucose concentration [25]. In patients with type 2 diabetes, PLA-M [26,27] and platelet-polymorphonuclear aggregates [27] were increased compared with healthy people and also correlated with glucose and triglycerides levels in plasma [26]. Similarly, in a population with type 1 diabetes, the levels of PLA-M [27,72,73] and platelet-polymorphonuclear aggregates were higher compared with the lack of diabetes [27]. In diabetes 1, PLA-M correlated with concentrations of glycated haemoglobin (HbA1c), triglycerides, total cholesterol (TC), and low-density lipoprotein (LDL) [72]. Concurrently, PLA-Ly and PLA-G were increased in insulin-dependent diabetes compared with healthy populations [74]. Cardiovascular risk increases with increasing plasma glucose concentrations [75], thus PLA-LyTh, PLA-M and platelet-polymorphonuclear aggregates correlating with glucose level could be considered a biomarker for cardiovascular risk stratification in patients with diabetes or metabolic syndrome [25,26,27]. 

An increased concentration of PLA-M, PLA-N, and PLA-Ly was noticed in hypercholesterolemia compared with a lack of dyslipidaemia [28,76]. What is more, PLA-M positively correlated with TC and LDL independent of dyslipidaemia. PLA-M or PLA-N negatively correlated with high-density lipoprotein (HDL) in hyperlipidaemic populations or healthy people, respectively [28]. 

The effect of lipid-lowering treatment on PLA is disputable. It seems to be dependent on concomitant states, leading to a decrease of this biomarker compared with placebo in patients with acute coronary syndromes [77] and an animal model of congestive heart failure [39], but in populations with coronary artery disease and impaired glucose tolerance or type 2 diabetes no changes compared with lack of treatment were observed [78]. 

Interestingly, item diet influences PLA percentage. In in vitro models, gallic acids occurring in red wines, tea, plants [79], and flavonolignans from the plant *Silybum marianum* lowered PLA level compared with a lack of supplementation [80]. In similar conditions, anthocyanins presenting in berry fruits and their metabolites decreased PLA-M and PLA-N compared with the lack of anthocyanins [81]. In mice, alfrutamide and caffedymide from plants like cocoa, coffee tree or garlic, inhibited PLA formation compared with lack of compounds from plants [40]. In people, supplementation of ω-3 fatty acids decreased PLA concentration compared with a lack of supplementation [82]. 

On the other hand, exhaustive exercises were connected with higher PLA levels [74,83,84,85,86]. Still, less intensified strain led to gentler PLA enlargement [83] and suggested that only light workouts could benefit health. 

To our best knowledge, no trials assess the influence of enlarged alcohol intake, decreased strength, or low education level, but other risk factors have a special connection with PLA.

### 3.2. Platelet–Leucocyte Aggregates in Chronic Coronary Syndromes

It was reported that the concentrations of PLA or, more specifically, PLA-M were higher in patients with CAD than in healthy controls. Besides, platelets from CAD patients form PLA-M more intensely in reaction to small concentrations of ADP or TRAP [87]. Other studies corroborate these findings [30,88]. Whether levels of PLA correlate with the severity of CAD is poorly documented. Two studies found no association between PLA and functional relevance of coronary stenosis quantified with fractional flow reserve (FFR) or several diseased vessels [89,90]. Nevertheless, one had a small sample size [89], and both assessed only the overall number of PLA-M [89,90], whereas PLA-M are heterogeneous concerning the phenotypes of monocytes [74]. In one study, a higher count of M2 containing PLA-M indicated a diffuse form of CAD. PLA-M, including M2, also negatively correlated with impaired endothelium-dependent vasodilation in these patients (the effect was stable over a 12-month observation), pointing to a possible background or the result of more advanced lesions [91]. Another study also demonstrated a positive correlation between endothelial dysfunction in coronary vessels (manifesting as paradoxical vasoconstriction in response to increased coronary flow) and PLA-M concentration in the context of CAD [90]. These findings encourage research into the specific roles of PLA-M and their subtypes in atherogenesis and as a potential marker of the extent of stable CAD.

More severe CAD in patients undergoing revascularization and valve replacement was linked with worse outcomes [92,93]. In a recent study high preprocedural M2 and CD-11b were independently associated with 3-month mortality after transcatheter aortic valve implantation. CD11b correlated with PLA-M (PLA-M subsets were not assessed) [94]. The interplay between CAD, PLA and prognosis after cardiac procedures remains to be understood better.

### 3.3. Changes in Platelet–Leucocyte Aggregates after Coronary Artery Bypass Grafting

The use of cardiopulmonary bypass during CABG and the surgery itself results in activation of platelets and leukocytes as reflected by amounts of P-selectin-positive platelets, CD11b positive leukocytes, platelet aggregation, and PLA. Ensuing in-pump aggregation of platelets results in their depletion immediately after the procedure, but their level culminates again on day 7—PLA amount also peaks at this time [95], stressing the role of platelets in initiating interaction with leukocytes. A recent study demonstrated increased platelet reactivity to agonists (ADP, thrombin, thromboxane analogue) up to 3 months after elective CABG in patients with stable angina compared with their state before operation. All PLA subtypes were elevated at 1 month, whereas at 3 months, PLA-Ly were elevated, but PLA-M and PLA-N were decreased compared with the baseline statement [96]. The significance of these findings for short- and long-term prognosis needs to be elucidated by further research.

We found no studies investigating the relationship between PLA formation in the post-CABG period and the patient outcome. Nevertheless, preoperative PLA was predictive of acute kidney injury (AKI) and 3-year risk of major adverse events in a cohort of surgical valve replacement due to rheumatic heart disease. The authors estimated that the optimal PLA cut-off to identify at-risk populations was 6.8%—patients with higher values before the operation were nearly 18 times more likely to suffer from AKI [97,98]. Cardiac surgery-associated acute kidney injury (CSA-AKI) is a complication common also in CABG patients [99]. However, its association with baseline PLA has not been studied so far. The advantage of PLA in predicting CSA-AKI is assessing the risk even before the procedure. In contrast, other known markers, such as neutrophil gelatinase-associated lipocalin, cystatin C, kidney injury molecule 1, or IL-18 are dependent on already existing damage to the kidney [100]. Identifying vulnerable patients before the operation might allow designing suitable interventions to reduce the additional risk. A certain type of exercise regime reduced PLA-M in post-CABG patients [101]. It remains an interesting question as to whether similar actions before cardiac surgery would effectively decrease the risk of AKI.

### 3.4. Platelet–Leucocyte Aggregates in Acute Coronary Syndromes

There are several ways by which PLA might contribute to ACS pathophysiology. The greatest platelet aggregability and concentrations of various coagulation markers are connected with morning hours and the start of activity [102,103], when blood catecholamines level and sympathetic tone are also high [104,105]. This is thought to play a role in the increased rates of cardiovascular events in the morning [105]. Anti-adrenergic medications lower PLA-M and PLA-N formation both in circulation and in response to ADP [106]. It suggests that PLA generation might be enhanced in the morning due to adrenergic signalling. Through PLA-M platelets induce cytokines [107] and tissue factor (TF) production in monocytes [108,109], an important source of microvesicle-borne TF, which promotes fibrin accumulation at the site of thrombus formation [110]. Disrupted P-selectin—PSGL-1 signalling led to reduced fibrin deposition within thrombus [110] and impaired aggregation of whole blood [61]. On monocytes, PLA-M also increased Mac-1 [3], which is involved in thrombosis and elevated after AMI [111]. In a mice model of carotid artery thrombosis, the lack of Mac-1 significantly prolonged time to occlusion [112]. In one study administration of abciximab to AMI patients undergoing PCI reduced both PLA-M and monocyte Mac-1 expression 24 and 72 hours (h) after the event [3]. Another important mechanism is the ability of platelets in PLA-N to cause the release of NETs from neutrophils [64,66]. Recently, interactions between platelets and leukocytes were even proposed as a possible pharmacological target to prevent atherothrombosis [113].

Among patients presenting with chest pain admitted to the emergency department, those undergoing acute myocardial infarction had a significantly higher level of PLA-M than the remaining non-infarction group [114,115]. However, this effect was not reported uniformly [116]. The evidence suggests that aggregate formation happens in the early myocardial infarction phase, when platelets are activated by ruptured plaque [115]. PLA-M elevation within 4 h from the onset of symptoms was over 1.5 times greater than between 4 and 8 h [114]. Additionally, intermediate monocytes, which are also candidates for AMI biomarkers, were raised due to monocyte–platelet interactions [117]. This means that PLA could be an even earlier marker of AMI. One small study showed that elevated PLA-M had strong discriminative power for AMI as early as 2 h from the onset of suspicious symptoms [118].

Total PLA-M and intermediate monocytes and their complexes with platelets were significantly higher on admission in patients with UA than stable coronary disease controls. These parameters correlated with the Global Registry of Acute Coronary Events (GRACE) score (which classifies patients into low- and intermediate-to-high risk) [119,120].

Additionally, patients with UA and non-ST elevation myocardial infarction (NSTEMI) predisposed for the relapse of ischemia could be distinguished based on the intensity of PLA-N formation in response to stimulation with TRAP. However, baseline levels of PLA-N were comparable between the groups [121]. This example stresses that a momentary level of PLA and the capacity for their formation may be of diagnostic or prognostic value as it testifies to the strength of inflammatory and atherogenic response.

### 3.5. Changes in Platelet–Leucocyte Aggregates after Percutaneous Coronary Intervention

Both short- and more distant-term prognoses after ST-elevation myocardial infarction (STEMI) treatment with PCI were associated with the intermediate monocytes at admission. It was observed that the concentration of PLA-M involving intermediate monocytes was independently associated with in-hospital complications (including left ventricle aneurysm, massive coronary thrombosis, and acute heart failure) over 7 days post event [34]. Similarly, it was reported that both intermediate monocytes and corresponding PLA-M, measured on day 2 following AMI (and standard treatment), independently predicted the 2-year incidence of major adverse cardiovascular events defined as cardiovascular death, nonfatal ischemic stroke, recurrent myocardial infarction, repeat revascularization, or rehospitalization for heart failure [122].

Evidence accumulates that elevated PLA in patients with STEMI may predict unsuccessful reperfusion after PCI termed coronary microvascular obstruction (MVO) or no-reflow phenomenon. Depending on the assessment method it was reported to complicate 2.3–56.9% of such cases, substantially increasing both early and late mortality [123,124,125]. One study found that the MVO group (MVO defined here as thrombolysis in myocardial infarction (TIMI) flow less than two or TIMI equal to three and a myocardial blush grade less than three despite residual stenosis less than 20%) compared with the successful reperfusion group had elevated PLA-M and their platelets expressed more activation markers in response to ADP, revealing a greater capacity for aggregation. Of note, those differences persisted at 1-month follow-up, despite dual antiplatelet therapy (DAPT) [126]. The authors suggested that these differences might underlie unfavourable outcomes associated with MVO [126]. In another study PLA, PLA-M, and PLA-N, but not PLA-Ly, were significantly associated with poor coronary perfusion in patients with STEMI treated with PCI within 12 h since the onset of symptoms [127]. Others also reported similar findings [128]. Although consistent, all the above studies included relatively few subjects and relied mainly on the angiographic diagnosis of MVO; we found no studies exploring the relationship between PLA and MVO defined based on cardiac magnetic resonance imaging. Hence, the continued effort to clarify the importance of PLA for MVO and no-reflow phenomenon should be warranted.

### 3.6. Treatment with β-Blockers in Acute Myocardial Infarction

It was observed that β-blockers, used chronically before AMI or in an early stage of the event improve the prognosis after STEMI [129]. Due to inhibition of corresponding receptors on leukocytes (and probably also platelets), the use of β-blockers was associated with reduced PLA-N and PLA-M formation, less inflammation, less microvascular obstruction, and accordingly smaller infarct size [106,130]. Metoprolol limited infarct size in METOCARD-CNIC trial (Effect of Metoprolol in Cardioprotection During an Acute Myocardial Infarction) but did not show significant improvements in a similar EARLY-BAMI trial (early-beta blocker administration before reperfusion primary PCI in patients with ST-elevation myocardial infarction). However, the studies differed substantially in time from diagnosis to administration, and the effect of β-blockers strongly correlated with timing, which might explain the lack of effect in the EARLY-BAMI study (median of 10 min in METOCARD-CNIC) [131]. These observations once again underlie the connection between the PLA levels and outcomes after acute coronary events.

### 3.7. Monitoring Antiplatelet Treatment

Antiplatelet therapy, consisting of acetylsalicylic acid (ASA) alone or with a P2Y12-inhibitor, constitutes a cornerstone of CVD treatment [132,133,134,135]. GP IIb/IIIa-blocker administration is advantageous in AMI with complications such as thrombosis or no-reflow [133,135]. In turn, prostacyclin analogues used in patients with pulmonary hypertension to dilate pulmonary vessels, also inhibit platelet activation [136]. In monitoring platelet function, P-selectin was considered the gold standard [137], but PLA turned out to be much more sensitive [115]. Hence, they seem to be promising candidates for a biomarker in antiplatelet treatment monitoring.

#### 3.7.1. Acetylsalicylic Acid in Monotherapy

The influence of ASA on PLA is not well stated. In in vitro trial, ASA was added to blood samples from half of 12 healthy participants, and there was no effect of this medicament on PLA-M and PLA-N, either in resting and activated by ADP or PAF specimens compared with an absence of medication [138]. Similar results were observed during oral intake of ASA. In 12 healthy men treated with 75 milligrams (mg) ASA daily for 10 days, there was no inhibition of PLA-M and PLA-N formation after stimulation with ADP compared with state before medicine administration [139]. Likewise, ASA did not inhibit spontaneous and induced by ADP, thrombin or PAF PLA, PLA-M, PLA-N, and PLA-Ly formation in 15 healthy men treated during a week in dose 75 mg and 500 mg for next 7 days compared with set point [140]. On the other hand, PLA-M percentage decreased in the healthy population of 40 after 10 days of 150 mg ASA intake compared with state before treatment, which suggested the necessity of more extended therapy with higher dose and possibly higher sensitivity of PLA-M as a marker than other PLA subtypes, but further studies are required due to the small group of participants [141]. The results from research in pathologies are inhomogeneous. In mice with temporal carotid artery strangulation treated with ASA, the percentage of PLA were increased in resting samples, and there were no changes after activation compared with placebo. However, in the same conditions, PLA as platelet to leucocyte ratio was decreased both with and without stimulation [142]. In another ischemia model, with an after-reperfusion effect on intestinal microcirculation in mice, there were less PLA after ASA intake than placebo [143]. Contrarily, there was no effect of 150 mg ASA prescription for 10 days on PLA in 63 patients directly after stroke [141]. Others reported that in patients who suffered a stroke within the previous 6 months, taking 81–650 mg of ASA daily for at least 1 month preceding testing was associated with significantly lower PLA compared with those who failed to take ASA at that time [144]. Subsequent studies are necessary to define the impact of ASA on PLA in particular diseases.

#### 3.7.2. P2Y12-Receptor Antagonists

Clopidogrel, prasugrel, ticagrelor, and cangrelor are enumerated as available in clinical practice as P2Y12 inhibitors [145]. In mice treated with clopidogrel, platelet-polymorphonuclear aggregates formation after stimulation with ADP or thrombin was lower than without this medicament. This effect was dependent on platelets. Mixing platelets from untreated mice with polymorphonuclears from mice treated with clopidogrel did not influence the creation of platelet-polymorphonuclear aggregates. Likewise, in whole blood drawn from healthy people and then incubated with active metabolite of clopidogrel, less platelet-polymorphonuclear aggregates were observed after stimulation with ADP or PAR agonist than without any medicaments. Clopidogrel also decreased the formation of reactive oxygen species by human polymorphonuclears incubated with platelets subjected to clopidogrel action [146]. Despite the relationship between clopidogrel and platelets, the mechanism of clopidogrel’s influence on PLA is not dependent on P-selection. In the human population with a deficient variant of P-selectin, clopidogrel inhibited PLA-M formation like in a group with accurate P-selectin [147]. In a clinical condition of ACS, clopidogrel successfully restored PLA-M and PLA-N to concentration nearly equal to those in healthy people and arrested the creation of PLA completely during ADP stimulation and to 50% after adding TRAP [148]. The impact of clopidogrel on PLA was stable during 30 days of observation [149].

On the other hand, patients with CCS and myocardial ischemia defined as FFR ≤ 0.75 treated with clopidogrel had a lower percentage of PLA-N than the population without ischemia. A similar trend occurred for PLA-M. This observation authors determine as paradoxical and explained by higher clearance of PLA, sequestration of leucocytes to the endothelium and migration in the direction of deeper located tissue [89]. 

The active metabolite of prasugrel, administrated orally for mice, inhibited connections between platelets and polymorphonuclears after stimulation [150]. Following combining prasugrel with blood from healthy volunteers with ADP or TRAP, this P2Y12 inhibitor contained platelet-polymorphonuclear aggregates [150], PLA-M, and PLA-N [151].

Similarly, the active form of cangrelor added in vitro [151] prevents an increase of PLA-M and PLA-N after stimulation with ADP [139]. Two other P2Y12 inhibitors reported in the literature but not applied in clinal practice presented variable action in adequate condition. AR-C69331 behaved like activated cangrelor [151,152], but the second one, AR-C69931, had no impact on PLA-M and PLA-N [115]. 

The pleiotropic role of P2Y12 inhibitors was previously described primarily in the context of supplementary impact on the immune system [153,154]. PLA are discussed as a critical mediator of anti-inflammatory action in this group of medicaments. In a mice model of sepsis, a lack of P2Y12 receptors caused lower PLA levels than in a population expressing P2Y12 [12]. On the other hand, clopidogrel inhibited PLA formation in mice with endotoxemia [155] or induced hypertension [156]. Additionally, an active form of prasugrel protected against platelet-activator-induced platelet polymorphonuclear formation in mice with endotoxic shock [150]. In healthy people treated with ticagrelor, PLA-M production was lower after in vitro exposure to toll-like receptor 2 and 4 (TLR-2, TLR-4) agonists than in the placebo group [157]. Ticagrelor significantly decreased PLA in patients with pneumonia after 24 h of treatment compared with their state before this pharmaceutical administration. Additionally, a higher level of PLA at baseline was connected with a more expressed effect of medicine [158].

#### 3.7.3. Acetylsalicylic Acid in Comparison with P2Y12-Receptor Antagonists

As cited above, it was proven that the P2Y12 antagonists decreased PLA level [139,146,148,150,151,152], but there is no direct comparison with ASA. However, in patients who had taken ASA or clopidogrel with a break of treatment for operation, the effect of ASA was more stable than clopidogrel during the lack of antiplatelet therapy [159].

#### 3.7.4. Dual Antiplatelet Therapy

DAPT consists of ASA, and a P2Y12 inhibitor represents a baseline of treatment after PCI because of its efficacy in preventing recurrent thrombotic events, including stent thrombosis [160]. In in vitro assessment, ASA with AR-C66931 as P2Y12 antagonist did not influence PLA-M and PLA-N formation [115]. Nevertheless, in patients with ACS after PCI, combining ASA with clopidogrel or cangrelor prevented PLA-M and PLA-N stirring, and this action peaked when combing all three medical preparations, but data about bleeding complications were not reported [139]. The superiority of combination ASA with clopidogrel over ASA was observed even in a population with lower responsiveness to clopidogrel. In patients with ischemic stroke and lessened function of CYP2C19*2 alleles (decreasing response to clopidogrel), ASA coupled with clopidogrel were more potent in lowering PLA than was ASA in monotherapy [161]. In comparison with monotherapy with ASA, DAPT turned out to be more successful in PLA inhibition in patients with CCS (ASA with clopidogrel) [162], unstable angina (ASA with prasugrel) [163], and ischemic stroke (ASA with clopidogrel) [164]. Prasugrel is taken into consideration as the best P2Y12 inhibitor for DAPT according to its impact on PLA. It was observed that, in patients with CCS, PLA was similar at baseline to ASA with clopidogrel or prasugrel. However, ASA with prasugrel was significantly more potent in reducing PLA generating while stimulated with agonists than ASA with clopidogrel, and this effect was maintained through 29 days of follow up [165].

#### 3.7.5. GP IIb/IIIa Inhibitors

Abciximab, eptifibatide, and tirofiban are used in clinical practice as medicaments inhibiting GP IIb/IIIa [135,136]. The impact of GP IIb/IIIa inhibitors on PLA is discussed and depends on subtypes of PLA, medicament, type of platelets stimulation, and conditions like temperature [166,167,168,169,170,171]. Abciximab decreased PLA production after douching by blood from healthy donors in a porcine model of damaged vessels compared with placebo [166]. Similarly, it was observed that less PLA-N in the blood from healthy humans when combined with non-peptide GP IIb/IIIa antagonist-SR121566 after stimulation with PAF, but SR121566 also led to an increase of PLA-N after ADP or adding TRAP compared with the absence of medicaments [167]. Likewise, eptifibatide increased mainly PLA-M and PLA-N after the exposure of blood from healthy volunteers to collagen compared with lack of pharmaceuticals [168]. In hypothermia (18 °C), eptifibatide elevated PLA-G creation in blood from healthy people stimulated with ADP compared with normothermia (37 °C) [169,170]. On the other hand, PLA-N levels did not change in blood from healthy populations after adding tirofiban and ADP or TRAP [171]. Interestingly, there were no changes in PLA-G and PLA-M in blood from healthy donors mixed with tirofiban in hypothermia compared with normothermia [170]. Higher PLA levels after exposure to GP IIb/IIIa are explained by the blocking of platelet-to-platelet binding and, thereby, more opportunity for platelet–leucocyte interactions [167,168].

There was also research conducted with patients with AMI treated orally with a GP IIb/IIIa inhibitor, and the results were more consistent than in vitro trials [3,166,167,168,169,170,171,172]. Decreased PLA level was observed during intake of abciximab combined with heparin and DAPT and abciximab or tirofiban (more pronounced effect) in conjunction with ASA, heparin, and thrombolysis compared with the absence of a GP IIb/IIIa inhibitor [3,172]. 

#### 3.7.6. Prostacyclin Analogues

Prostacyclin analogues have evidenced impact as an inhibitor of platelet reactivity and thrombus formation in pulmonary hypertension [173]. Epoprostenol, as a medicament from this group of pharmaceuticals, inhibited PLA creation after adding whole blood from healthy people and stimulation with ADP compared with ASA or cangrelor or prostaglandin E1 [174].

Results of PLA investigation in various clinical states are summarized in Table 1.

## 4. Methods of Measurement

### 4.1. Conventional Flow Cytometry

**Figure 3:**
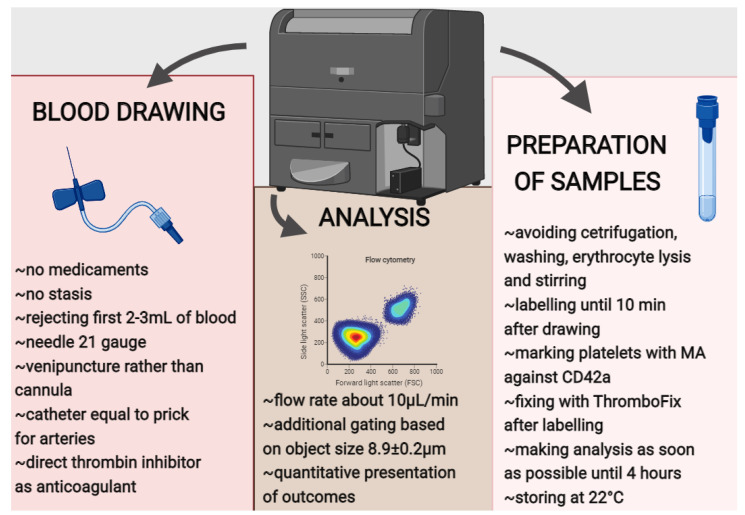
Recommendations regarding blood withdrawal, sample handling and platelet–leucocyte aggregates for measurement with conventional flow cytometry. Abbreviations: CD—cluster of differentiation; MA—monoclonal antibodies; min—minute; mL—millilitre; µm—micrometre; µL/min—microlitre per minute; °C—degrees Celsius.

CFC is a method of choice for PLA assessment [24] and very handy for clinical practice [36,37]. The main advantages of CFC are no requirement for very advanced machine [24], availability in laboratories [36], easy access and a tiny amount (~5 microlitres [µL]) of whole blood as research material [47], quick measurement, high sensitivity, precision of assay [24], one-step detection, and quantifying PLA with distinction of leucocyte subsets, as well as assessment of platelet or leucocyte activity [31]. On the other hand, a very detailed test involves exceptional precision in handling and avoiding artifacts resulting from platelets’ activation post drawing [175]. Figure 3 shows the possible traps in detecting and quantifying PLA with CFC. There are many published protocols for measuring PLA with CFC [4,36,37,47], but there are no standardized roles as in other biomarkers, such as EVs [176]. There is also a lack of regular extents in health and pathologies. These facts prohibit the effective comparing of results from various trials and the wide application of PLA as a marker in clinical practice. 

Whole blood was usually taken for PLA assessment with CFC [4,24,36,37,46,47,50,175,177,178]. To the best of our knowledge, there is a lack of research proving the impact of applying a tourniquet, using a needle with a low diameter, or not rejecting the first blood portion on PLA level. For comparison in EVs also dependent on platelet or leucocyte, stasis, a needle diameter below 21 gauge and including the first 2–3 millilitres (mL) of blood to samples are not recommended [176]. On this account, it seems to be justified to avoid these activities during blood drawing, according to some authors [36,37,46,50,175]. What is more, it was proved that clean antecubital venepuncture, rather than intravenous cannula, is the best way for samples to be sourced because it was shown not to cause an increase of PLA level, even during repeated blood drawing [175].

On the other hand, arterial blood sampling with a catheter and a prick is equally safe for PLA assessment [178]. Sodium citrate or EDTA decreased, but heparin increased PLA level. Hence, direct thrombin inhibitors are recommended as anticoagulants [175].

Some authors reported using centrifugation and washing of the biological material, but these processes lead to cell loss and additional PLA formation [50]. For that reason, they are not recommended. In avoiding this process, the first step, most often, is marking platelets and leucocytes [4,36,50,175,179]. However, some authors fixed samples before following procedures [37,50]; however, latency over 10 min in labelling decreased repeatability in the number of PLA [175]. This reaction was weaker when sodium citrate was used as an anticoagulant instead of a direct thrombin inhibitor [175]. Fluorescing substances were applied for designating monoclonal antibodies (MA) connected with PLA [36]. It is important to mark cells with MA that do not influence molecules participating in the adhesion of leucocytes to platelets, especially when stimulation of PLA formation is planned. On this account, MA against GP Ib/IX (CD42) had the edge over against GP IIb/IIIa (CD41/CD61) [50]. Nevertheless, platelets can lose GP Ib (CD42b) from their surface [46,50]. For that reason, it is recommended to use MA against GPIX (CD42a) for labelling these blood-morphotic elements [46]. Except using MA for red-tagging platelets, at least one other MA specific for all leucocytes (CD45) [4] or their particular subtype [50] is required. One-step marking is possible with more than 2 MAs. CFC enables, additionally, the distinction of leucocyte subsets: lymphocytes B (CD19), lymphocytes T (CD3) helper (CD4) and cytotoxic (CD8), NK-cells (CD56), granulocytes (CD15), monocytes (CD14), neutrophils (CD16), eosinophils (lack of CD16) or assessment of platelet (CD62P), and leucocyte (CD11b) activation [4,25,36,37,46,47,50,74].

Although some authors fixed samples before labelling [36,50,179], most researchers followed steps in inverse order [4,36,46,50,175,179]. In unfixed specimens, at room temperature, the PLA level started increasing after 0.5 to 1 h. It is possible to use formaldehyde or paraformaldehyde to fix samples, but these substances could cause artefactual PLA formation [4,50]. In addition, their effect on blood stability is uncertain because, after using them, PLA increase was observed after 20 minutes (min) to 72 h [47]. After fixing samples with FACS-Lyse, PLA were stable at 4 °C over 24 h, but the percentage of PLA was not assessed with an unfixed control [175]. It is possible to use CellFix [36,180], but an increase of aggregates, at both 4 and 25 °C during 6 h in patients with CVD and healthy people, was observed [180]. On the other hand, ThromboFix protected against PLA formation for a week but guarded in vivo-ensuing PLA only for 4 h [181]. Some scholars chose erythrocytes lysis [4,50,175], but outcomes were inconsistent [50,175]. 

Many authors exploited in vivo stimulation to explore the ability of PLA formation in samples enhanced by platelets, leucocytes, or the activation of both [4,36,37,46,47,50,179]; as stimulation, substances like ADP, TRAP, collagen, epinephrine, LPS, TNFα, thrombin, PAF, and fMLP were used [36,50]. This step is not necessary but could yield extra information. It is suggested to store samples at 22 °C and avoiding stirring [46]. 

After preparation, probes are analysed by CFC. The flux speed is recommended to be as slow as possible (about 10 µL/min) because, during faster flow, more PLA were shown to form [182]. To detect PLA with CFC, it is essential to find platelets tethered with leucocytes. For this goal, gating based on fluorescence was applied [4]. Additionally, to avoid artifacts consequent to detecting EVs exposing the same markers, gating was employed based on objects’ sizes [36]. 

CFC permits quantifying PLA and its subsets [4], but it is not possible to count directly the number of platelets using them [24]. The amount of PLA can be shown as a percentage of leucocytes (or subsets thereof, respectively) conjugated with platelets [4,46,177] or a percentage of platelet aggregates containing leucocytes [179]. Some authors used mean fluorescence index (MFI) as a gauge of PLA level [177], but this is only a semi-quantitative method [24,182]. Other researchers extrapolate the number of platelets tethering to leucocytes based on fluorescing, but this could vary from the percentage of PLA [141].

Despite various advantages of CFC in PLA assessment, the main obstacle to overcome is the lack of a unified protocol for drawing, handling, processing samples, presenting the results thereof, and a range of norms. This state precludes comparison between outcomes from various laboratories and clinical applications of CFC.

### 4.2. Imaging Flow Cytometry

IFC combines the advantages of CFC and fluorescence microscopy. It is possible to differentiate the proximity of platelets and leucocytes from PLA more effectively than with CFC. What is more, IFC enables higher throughput without significant over-detection of PLA [182].

### 4.3. Microscopy

Light microscopy also could be used to assess PLA [4,37]. Lower levels of PLA were observed in a sample during microscopic observation than with CFC measuring of the same sample [4], but a proportion of PLA subtypes correlated between these two methods [37]. The reason for this difference in observed PLA concentrations could be the lack of having excluded EVs [4], the random closeness of platelets and leucocytes without tethering [182], or trogocytosis [4] during assessment with CFC. Light microscopy also facilitates the denotation of PLA placement in tissue, but the picture is static. Alternatively, intravital microscopy, including wide-field epifluorescence, multiphoton, spinning-disk, or scanning confocal microscopy, allows assessing the live dynamics of platelet–leucocyte interaction in animal models [24,183]. Despite this advantage, microscopy is more suitable for experimental research than for clinical application [24], because it is less effective and more time-consuming than CFC [182].

## 5. Summary and Future Possibilities

Historically, over 40 years of research of PLA has provided increasingly more information about them, but still, it is not known how exactly they form, nor their detailed function in the pathogenesis of atherosclerosis and, thus, in CVD. The main reason for this missing information is the lack of a uniform protocol for PLA assessment using flow cytometry as the most common tool in clinical trials. On this account, researchers apply a variety of methods, and their results, in some cases, significantly diverge from others. Despite this, the available data allow us to consider PLA as the most sensitive marker of platelet activation. This development opens a wide extent of purpose. PLA could help to diagnose CVD, predict complications after PCI or CABG, monitor the response to antiplatelet therapy, and predict the overall risk of future cardiovascular events. As a biomarker dependent on platelets and leucocytes, they could also help discover and assess the pleiotropic results of treatment. In addition, PLA have a chance to be the target of new medications. Further studies are required, first of all, to introduce a consistent protocol for PLA assessments and the reporting of results thereof. Validation and the determination of normal ranges are also essential.

Finally, as mentioned before, PLA are now being widely investigated as potential cardiovascular complication markers in COVID-19. It is probably the most up-to-date field of interest in PLA research. Although SARS-CoV-2 vaccines induce spike protein overexpression, vaccination with BNT162b2 does not alter platelet protein expression, and reactivity [184] but SARS-CoV-2 infection induces PLA formation rather than platelet–platelet aggregates [185], and PLA are accused of forming blood clots in severe COVID-19 [186]. Altogether, they create a new concept of PLA’ pathomechanism in COVID-19 cardiovascular complications, which deserves separate review.

## Figures and Tables

**Table 1:** Platelet-leukocyte aggregates changes in various clinical condition.

Clinical Condition	No. of Patients	Control Group	Outcome	Reference
Clinical Studies				
CVD	yes, N = 345	no, N = 64	CVD cohort, including patients with CAD, PAD, carotid artery stenosis and abdominal aortic aneurysm, had significantly higher PLA-M compared with healthy controls (both groups received ASA). The association was true for each CVD separately, but after multivariant analysis it remained significant only for PAD. Patients with critical limb ischemia had significantly higher PLA-M than other PAD patients.	Allen et al., 2019 [19]
peripheral artery disease	critical limb ischemia, N = 20	intermittent claudication, N = 45 healthy, N = 20	PLA correlated with the disease severity. Critical limb ischemia was associated with significantly higher PLA-M with intermediate or non-classical monocytes, total PLA-M and PLA-N.	Dopheide et al., 2016 [20]
atrial fibrillation	with left atrium thrombus, N = 27	without left atrium thrombus, N = 80	Among patients assessed with transoesophageal echocardiography before electric cardioversion/ablation PLA-M over 170 cells/μl independently predicted left atrium thrombus with sensitivity of 93%.	Pfluecke et al., 2016 [21]
venous thromboembolism	VTE, N = 13	no VTE, N = 19	Patients who suffered VTE 30 days following orthopaedic surgery had significantly elevated PLA-M 24 h after the procedure compared with the group without this complication.	Shin et al., 2016 [22]
abdominal aortic aneurysm	AAA, N = 41	no AAA, N = 38	Although significantly more activated and newly released neutrophils circulated in blood of AAA group, it was not true for PLA-N or PLA-M.	Zagrapan et al., 2019 [23]
smoking	yes, N = 10	no, N = 10	PLA-M, PLA-N and PLA-Ly were significantly higher in smokers.	Badrnya et al., 2014 [41]
smoking	yes, N = 20	no, N = 20	Smokers had significantly higher PLA-M and PLA-N than controls. Three-weeks-long smoking cessation led to significantly reduced PLA-M formation.	Lupia et al., 2010 [42]
hypertension	yes, N = 22	no, N = 22	Hypertensive subjects had significantly higher PLA at baseline compared with controls. This effect persistent during 2 months’ observation despite the treatment with doxazosin and a trend towards pressure normalization.	Labios et al., 2006 [43]
depression	yes, N = 102	no, N = 44	Depressed patients had significantly higher PLA, PLA-M, PLA-G, than controls. This state persistent after 6 months of therapy despite concurrent improvement in platelet reactivity.	Morel-Kopp et al., 2009 [44]
exposure to biomass smoke	yes, N = 165	no, N = 155	PLA-M and platelet-polymorphonuclear aggregates were elevated in women cooking with biomass.	Ray et al., 2006 [45]
metabolic syndrome	yes, N = 18	no, N = 21	Metabolic syndrome was associated with elevated PLA-LyT and PLA-E.	Marques et al., 2019 [25]
type 2 diabetes mellitus	yes, N = 14	no, N = 14	Elevated PLA-M; its level correlated positively with plasma glucose and TG.	Patko et al., 2012 [26]
type 1 and type 2 diabetes mellitus	yes, N = 65	no, N = 25	Diabetic patients exhibited higher platelet–polymorphonuclear aggregates and PLA-M regardless of the disease type. Concentrations of aggregates were higher in diabetics with vascular lesions.	Elalamy et al., 2008 [27]
type 1 diabetes mellitus	yes, N = 35	no, N = 20	The patients had significantly higher PLA-M. Its level correlated with HbA1c, total cholesterol, LDL and TG.	Zahran et al., 2018 [72]
type 1 diabetes mellitus	T1D with microangiopathy, N = 20 T1D without microangiopathy, N = 19	healthy, N = 27	In T1D with microangiopathy baseline PLA-M and PLA-N were slightly higher that in controls. Total baseline PLA was comparable between all groups. TXA2 analogue-induced PLA formation was significantly greater in T1D subjects, whereas thrombin-induced PLA formation was significantly higher only in T1D with microangiopathy compared with controls.	Hu et al., 2004 [73]
type 1 diabetes mellitus, exercise	T1D, N = 16	healthy, N = 16	At rest T1D group showed higher PLA-G and PLA-Ly (spontaneous and TRAP-induced). Exercise significantly increased PLA-G, PLA-M, PLA-Ly in both groups; the effect of exercise was comparable between T1D and controls.	Hilberg et al., 2004 [74]
hyperlipidemia	yes, N = 8	no, N = 8	Hyperlipidaemic subjects had higher baseline PLA-M and PLA-N. Their PLA-M correlated positively with total cholesterol, LDL, and serum fibrinogen, and negatively with HDL.	Sener et al., 2005 [28]
primary hypercholesterolemia	yes, N = 22	no, N = 21	PLA-M, PLA-N and PLA-Ly were increased in PH compared with controls, which resulted in greater adhesiveness to arterial walls.	Collado et al., 2018 [76]
ACS	rosuvastatin, N = 21	placebo, N = 23	Administration of a high dose of rosuvastatin within 8 h of symptoms onset led to significantly lower PLA in rosuvastatin group compared with placebo at 8 and 24h after treatment.	Sexton et al., 2015 [77]
stable CAD with T2D/impaired glucose tolerance	simvastatin 80 mg, N = 16 ezetimibe 10 mg and simvastatin 10 mg, N = 16	without lipid-lowering treatment, N = 32	Patients received their assigned medication once daily for six weeks-no significant effect on baseline PLA was found.	Malmstrom et al., 2009 [78]
gallic acid	with gallic acid, N = 5	without gallic acid, N = 5	In vitro gallic acid significantly and dose-dependently decreased stimulation-induced (ADP, TXA2 analogue) PLA-M and PLA-G formation in blood from healthy subjects.	Chang et al., 2012 [79]
flavonolignans	with silychristin or silybin or silydianin, each N = 12	no flavonolignan, N = 12	In whole blood from healthy volunteers silychristin and silybin (but not silydianin) significantly and dose-dependently reduced PLA formation induced with IL-1β.	Bijak et al., 2017 [80]
anthocyanins	with 1 of 10 tested compounds, each N = 7	no anthocyanins added, N = 7	During in vitro ADP stimulation 2 of tested compounds (cyanidin-3-arabinoside, cyanidin-3-galactoside) significantly reduced PLA-N, ferulic acid reduced PLA-M and 4-hydroxybenzaldehyde reduced both.	Krga et al., 2018 [81]
cardiac surgery	supplementation, N = 7	no supplementation, N = 7	Preoperative supplementation of omega-3 fatty acids for 5 days resulted in significantly lower PLA on the day of operation and first post-operative day.	Iwase et al., 2014 [82]
exercise	after exercise, N = 20	before exercise, N = 20	PLA-G, PLA-M and PLA-Ly were significantly elevated after both moderate and strenuous exertion, although higher intensity caused significantly greater elevation of PLA-G and PLA-Ly.	Hilberg et al., 2008 [83]
exercise	after exercise, N = 21	before exercise, N = 21	PLA were significantly elevated after exertion; the effect was not abolished by enoxaparin nor argatroban administration. However, enoxaparin and argatroban did reduce PLA formation on thrombin or ADP stimulation post-exercise.	Li et al., 2007 [84]
exercise	after exercise, N = 14	before exercise, N = 14	PLA were significantly elevated after exertion, but percentage of PLA in regard to leukocyte count was similar. Increased PLA formation in response to exertion was not abolished by pretreatment with clopidogrel.	Perneby et al., 2004 [85]
exercise	MIH after exercise, N = 10 SIH after exercise, N = 10	MIH before exercise, N = 10 SIH before exercise, N = 10	After strenuous exercise PLA-E formation was significantly increased in response to LPS, shear stress and fMLP. Eight-weeks-long intermittent hypoxia regimen abolished this effect in both MIH and SIH group.	Wang et al., 2007 [86]
angiography for CAD evaluation	ACS, N = 125	No ACS, N = 437	PLA-M were significantly higher in ACS patients than those with and without angiographically-proven CAD. There was no association between baseline nor TRAP-induced PLA-M and adverse clinical outcomes (as defined in the study) in 2 years of observation.	Gremmel et al., 2016 [88]
	adverse clinical outcomes, N = 117	no adverse clinical outcomes, N = 445		
CAD	FFR(+), N = 75	FFR(-), N = 70	PLA-M did not significantly differ dependent on FFR status. Higher PLA-M associated with coronary vasomotor dysfunction independent of FFR status.	Di Serafino et al., 2014 [90]
	vasoconstrictors in endothelial function test, N = 10	vasodilators in endothelial function test, N = 20		
CAD	diffuse CAD, N = 50	healthy, N = 50 focal CAD, N = 40	PLA-M with M2 was significantly higher in the diffuse CAD group than the others; PLA-M with M2 independently and inversely correlated with endothelium-dependent vasodilation in patients with the diffuse disease.	Brown et al., 2018 [91]
CABG	after CABG, N = 15	before CABG, N = 15	PLA were increased for at least 1 week after the surgery and returned to baseline after 3 months. Immediately after CABG ADP- and TRAP-induced PLA formation was reduced, but at 1 week it was markedly increased. The effects were more evident for PLA-N and PLA-M.	Li et al., 2003 [95]
CABG for stable angina pectoris	after CABG, N = 54	before CABG, N = 54	One month after CABG PLA-M, PLA-N and PLA-Ly were significantly increased (unstimulated, ADP, thrombin, thromboxane analogue). At 3 months a significant decrease was observed in PLA-M (unstimulated, ADP, thromboxane analogue) and PLA-N (ADP, thromboxane analogue), whereas there was an increase for PLA-Ly (ADP).	Ivert et al. 2019 [96]
cardiac surgery (valve replacement for rheumatic heart disease)	AKI, N = 15	no AKI, N = 229	PLA in AKI group were significantly higher at all points throughout the perioperative period. High PLA (>6.8%) preoperatively entailed 18 times greater risk of AKI.	Yang et al., 2021 [97]
cardiac surgery (valve replacement for rheumatic heart disease)	high PLA, N = 151	low PLA, N = 93	Patients with high PLA (>6.8%) preoperatively, experienced significantly more major adverse events and perioperative complications than low PLA (<6.8%) group.	Liu et al., 2019 [98]
CABG	short-term intensive training after CABG, N = 19	conventional training after CABG, N = 21 healthy, N = 15	Strenuous exercise increased PLA-M and PLA-M subset with M1 in conventional training group but not in short-term intensive training group nor healthy controls.	Huang et al., 2017 [101]
AMI	AMI, N = 61	non-AMI chest pain, N = 150	In patients with chest pain PLA was significantly higher in AMI group. PLA level was the highest within 4 h from the onset of symptoms.	Furman et al., 2001 [114]
AMI	AMI, N = 9	non-AMI chest pain, N = 84 healthy, N = 10	Among patients with chest pain PLA-M were significantly higher in AMI group compared with non-AMI and control subjects.	Michelson et al., 2001 [115]
ACS	ACS, N = 43	non-ACS chest pain, N = 31	Among patients with chest pain PLA-M did not differ significantly between ACS and non-ACS subjects.	Levinas et al., 2012 [116]
AMI	AMI, N = 9	non-AMI chest pain, N = 21	Among patients suspected of AMI, those with actual AMI had significantly higher PLA-M. 100% of sensitivity and specificity for AMI with a PLA-M cut-off at 31.6%.	Lippi et al., 2007 [118]
UA	UA, N = 95	stable CAD, N = 30	UA patients had significantly higher total PLA-M, PLA-M with M2 and M3. Higher total PLA-M and PLA-M with M2 independently associated with intermediate-to-high risk by GRACE score.	Zeng et al., 2014 [119]
recurrent ischemia after UA/NSTEMI	yes, N = 7	no, N = 21	Patients who developed recurrent ischemia exhibited significantly greater PLA-N formation after adding TRAP. For every 1% increase in PLA-N the risk of recurrent ischemia rose about 7%.	Faraday et al., 2004 [121]
AMI treated with PCI	AMI, N = 31	healthy, N = 28	On admission AMI patients had higher PLA-M (in all subsets) than healthy controls. After adjustments higher PLA-M with M2 was significantly and independently associated with complications within 7 days of hospitalization in AMI patients.	Loguinova et al., 2018 [34]
AMI treated with PCI	STEMI, N = 100	stable CAD, N = 60 healthy controls, N = 35	PLA-M containing either M1 or M2 were significantly higher in STEMI group than in stable CAD at baseline. Higher accumulation of M2 and PLA-M with M2 on the seven days following AMI corresponded with the 2-years risk of serious cardiovascular adverse events.	Zhou et al., 2016 [122]
AMI treated with PCI	STEMI and successful reperfusion N = 35 STEMI and MVO, N = 13	stable CAD, N = 20	Baseline PLA-M were significantly higher in STEMI patients compared with stable CAD. MVO group had significantly higher PLA-M both on admission and 1-month post-treatment compared with good reperfusion group.	Aurigemma et al., 2014 [126]
AMI treated with PCI	poor perfusion, N = 28	good perfusion, N = 115	Patients with poor reperfusion after intervention had significantly higher PLA, PLA-M, PLA-N in aortic blood immediately after the procedure. PLA-N level showed independent correlation with sumSTR.	Huang et al., 2016 [127]
AMI treated with PCI	no-reflow, N = 19	successful reperfusion, N = 64	Patients with no-reflow after PCI had statistically greater preprocedural amount of PLA. High PLA appeared to be an independent risk factor for MVO.	Ren et al., 2016 [128]
ASA monotherapy	with, N = 6	without, N = 6	ASA did not affect spontaneous nor induced (with ADP, PAF) formation of PLA-N and PLA-M in vitro.	Zhao et al., 2001 [138]
ASA monotherapy	with, N = 15	without, N = 15	PLA amount did not differ before and after aspirin administration in healthy subjects.	Li et al., 2003 [140]
ASA monotherapy	with, N = 40	without, N = 40	Only PLA-M, but not PLA-G, amount differed significantly before and after ASA administration in healthy subjects. PLA formation in response to TRAP was not significantly affected.	Lukasik et al., 2011 [141]
clopidogrel treatment	Pro715-allele, N = 10 PSGL-1 B-allele, N = 5	without polymorphism, N = 10	PLA-M formation in response to ADP and TRAP was similarly reduced by clopidogrel both in controls and polymorphisms carriers.	Klinkhardt et al., 2005 [147]
clopidogrel for NSTEMI	NSTEMI on admission, N = 23	healthy, N = 20 NSTEMI group 24 h after clopidogrel, N = 23	NSTEMI patients had significantly higher PLA-M and PLA-N and enhanced PLA formation in response to ADP on admission. Clopidogrel counteracted both of these effects.	Xiao et al., 2004 [148]
coronary stenting	on clopidogrel after stenting, N = 51	before clopidogrel use and stenting, N = 51	Clopidogrel significantly reduced PLA formation in response to ADP, but not collagen. Nevertheless, PLA was significantly elevated for 30 days following the procedure.	Gurbel et al., 2002 [149]
P2Y12 and NHE-1 inhibitors treatment	with cariporide or AR-C 69331 or cariporide with AR-C 69331, each N = 8	no drug, N = 8	P2Y12 inhibitor AR-C69331 significantly limited PLA-M formation at normal and 7 pH, whereas NHE-1 inhibitor cariporide was only effective at 7 pH (conditions of NHE-1 activation). Combination of these agents had additive effect on PLA-M at 7 pH.	Klinkhardt et al., 2003 [152]
ticagrelor in healthy subjects	ticagrelor, N = 7	placebo, N = 7	Ticagrelor was associated with significant reduction in PLA-M formation in response to LPS and Pam3CSK4, but not spontaneous. Type of produced cytokines did not depend on ticagrelor use but seemed to differ due to receptor (TLR-2 or TLR-4).	Tunjungputri et al. 2014 [157]
pneumonia	ticagrelor, N = 30	placebo, N = 30	PLA decreased significantly in patients on ticagrelor in 24-h, while it increased in placebo group after the same time. Ticagrelor group had better forced expiratory volume and needed less oxygen supplementation after the treatment.	Sexton et al., 2018 [158]
chronic antiplatelet treatment	clopidogrel group, N = 15 aspirin group, N = 15	healthy, N = 15	5 days after 7 days’ break in antiplatelet therapy and aortic surgery PLA were significantly elevated in the clopidogrel group, but not in the aspirin group.	Le Manach et al., 2014 [159]
ischemic stroke	clopidogrel with ASA, N = 284	ASA monotherapy, N = 286	Patients who experienced early neurological deterioration had higher baseline PLA-N, PLA-M, PLA-Ly. Significantly lower PLA levels at 7–10 days of treatment with clopidogrel + ASA, but only in a group with functionally deficient CYP2C19*2	Yi et al., 2018 [161]
stable CAD	ASA with clopidogrel, N = 16	ASA with placebo, N = 15	ASA with clopidogrel group showed lesser PLA and PLA-M formation in response to ADP and thrombin.	Perneby et al., 2007 [162]
UA treated with PCI and DAPT	clopidogrel, N = 23 prasugrel, N = 22	before clopidogrel, N = 23 before prasugrel, N = 22	After 3 months, prasugrel group showed 40% decrease in ADP-induced PLA formation. There was a moderate negative correlation between ADP-induced PLA formation and flow-mediated dilation (measurement of endothelial function).	Rudolph et al., 2017 [163]
acute ischemic stroke	ASA with clopidogrel, N = 284	ASA monotherapy, N = 286	Patients receiving DAPT had significantly lower PLA-N, PLA-M and PLA-Ly after 30 days of treatment. DAPT reduced recurrence of stroke more effectively than ASA monotherapy.	Yi et al., 2014 [164]
stable CAD	prasugrel, N = 55	clopidogrel, N = 55	PLA-M formation induced with ADP was significantly decreased in prasugrel group compared with clopidogrel. The effect persisted at least one month after starting the treatment.	Braun et al., 2008 [165]
glycoprotein IIb/IIIa inhibitor treatment	with SR121566 or c7E3, each N = 5	without SR121566 and c7E3, each N = 5	In vitro SR121566 and c7E3 (glycoprotein IIb/IIIa inhibitors) significantly reduced PAF-induced PLA-N formation but led to augmented PLA-N formation in response to ADP and TRAP.	Hu et al., 2003 [167]
epifibatide treatment	with epifibatide, N = 10	without epifibatide, N = 10	Epifibatide significantly augmented collagen-induced formation of PLA-M (stronger effect) and PLA-N in whole blood from healthy volunteers.	Scholz et al., 2002 [168]
hypothermic conditions	with epifibatide at 18 °C, N = 10	with epifibatide at 37 °C, N = 10	When epifibatide was added, after ADP stimulation PLA-G were significantly elevated in hypothermic conditions (18 °C) compared with normothermia (37 °C).	Straub et al., 2005 [169]
hypothermic conditions	with tirofiban or epifibatide or abciximab, each N = 4	no drug, N = 4	In normothermia (37 °C) each of three drugs partially reduced ADP-induced PLA-M and PLA-N formation compared with control. However, this effect disappeared in hypothermic conditions (32 °C).	Frelinger et al., 2003 [170]
AMI	with reteplase or reteplase with abciximab or tenecteplase with tirofiban, each N = 5	before drug, each N = 5	ADP-induced PLA formation was significantly reduced in patients treated with reteplase with abciximab as well as tenecteplase with tirofiban with the maximum effect at 2 h after administration.	Bertram et al., 2002 [172]
epoprostenol administration	epoprostenol, N = 5	PGE1 or ASA or cangrelor, each N = 4–6	Epoprostenol inhibited PLA-M and platelet-polymorphonuclear aggregates formation in blood from healthy subjects. In vitro concentration inhibiting 50% of the maximal response to stimulation (collagen with ADP) was smaller for epoprostenol (nanomolar range) than reference drugs in the study: PGE1, aspirin, cangrelor (micromolar range).	Tamburrelli et al., 2011 [174]
**Preclinical studies**				
CHF and statin treatment	CHF, N = 22 rats CHF with rosuvastatin, N = 15 rats	healthy, N = 15 rats	Rat model of CHF was characterized by significantly increased PLA compared with controls. Amount of PLA in this animal was normalized on rosuvastatin treatment.	Schafer et al., 2005 [39]
alfrutamide, caffedymine	with alfrutamide or caffedymine, each N = 5	without alfrutamide and caffedymine, each N = 5	In vitro and in vivo both compounds significantly reduced unstimulated PLA formation in blood from mice.	Park et al., 2016 [40]
temporary ligation of the common carotid artery	ASA, N = 11 rivaroxaban, N = 12 ASA with rivaroxaban, N = 6	placebo, N = 11	Without stimulation ASA treated groups had significantly higher PLA compared with controls. No treatment reduced effect of 2-MeSADP stimulation on PLA, but rivaroxaban eliminated stimulating effect of PAR4 agonist.	Mastenbroek et al., 2020 [142]
ischemia-reperfusion injury in intestinal microcirculation	cilostazol, N = 14 mice ASA, N = 14 mice	placebo, N = 14 mice	Both cilostazol and ASA groups showed reduced PLA in intestinal circulation on microscopic assessment. However, only cilostazol group exhibited less endothelial damage.	Iba et al., 2006 [143]
clopidogrel treatment	mice treated with clopidogrel, N = 15–20	untreated mice, N = 15–20	Ability to form platelet–polymorphonuclear aggregates in response to ADP or thrombin was reduced in blood from treated mice. Platelets from untreated animals combined with polymorphonuclear cells from treated animals gave results like in untreated animals. Pretreating human blood with active metabolite of clopidogrel significantly reduced platelet–polymorphonuclear aggregates formation induced by ADP or PAR-1 agonist.	Evangelista et al., 2005 [146]
	human blood pretreated with clopidogrel ^1^, N = 3	human blood, N = 3		
murine model of endotoxemic shock	prasugrel, N = 5	placebo, N = 5	In prasugrel group, platelet-polymorphonuclear aggregates formation was significantly reduced after stimulation with PAR-4 agonist peptide (only low concentrations) both after and without LPS injection.	Totani et al., 2012 [150]
murine model of endotoxemia	LPS with clopidogrel pretreatment, N = 10	placebo, N = 10 LPS, N = 10 clopidogrel, N = 10	LPS injection resulted in PLA-N elevation, but it was significantly lower in subjects pretreated with clopidogrel. Clopidogrel pretreatment group showed less histological change and less leukocyte and platelet infiltration in the lungs compared with LPS alone group.	Wang et al., 2019 [155]
murine model of hypertension and cardiac remodelling	with clopidogrel, N = 4	without clopidogrel, N = 4	High pressure (due to infusion of angiotensin II or phenylephrine) was associated with high PLA formation. Clopidogrel treatment counteracted this effect leading to a significant PLA reduction.	Jia et al., 2013 [156]

Abbreviations: 2-MeSADP—2-methylthioadenosine diphosphate; AAA—abdominal aortic aneurysm; ACS—acute coronary syndrome; ADP—adenosine diphosphate; AKI—acute kidney injury; AMI—acute myocardial infarction; ASA—acetylsalicylic acid; CABG—coronary artery bypass graft; CAD—coronary artery disease; CHF—congestive heart failure; CVD—cardiovascular diseases; DAPT—dual anti-platelet therapy; FFR—fractional flow reserve; FFR(+)—hemodynamically significant stenosis; FFR(-)—hemodynamically non-significant stenosis; fMLP—N-formyl-methionyl-leucyl-phenylalanine; GRACE—the Global Registry of Acute Coronary Events score; HbA1c—glycated haemoglobin; HDL—high-density lipoprotein; LDL—low-density lipoprotein; LPS—lipopolysaccharides; MIH—moderate induced hypoxia; MVO—coronary microvascular obstruction; M1—classical monocytes; M2—intermediate monocytes; M3—non-classical monocytes; NHE-1—sodium–hydrogen exchanger isoform 1; NSTEMI—non-ST elevation myocardial infarction; PAD—peripheral artery disease; PAF—platelet-activating factor; PAR-1,4—protease activated receptor 1,4; PCI—percutaneous coronary intervention; PLA—platelet-leukocyte aggregates; PLA-E—platelet-eosinophil aggregates; PLA-G—platelet-granulocyte aggregates; PLA-Ly—platelet-lymphocyte aggregates; PLA-LyT—platelet-lymphocyte T aggregates; PLA-M—platelet-monocyte aggregates; PLA-N—platelet-neutrophil aggregates; PGE1—prostaglandin E1; PH—primary hypercholesterolemia; SIH—severe induced hypoxia; STEMI—ST elevation myocardial infarction; sumSTR—sum-ST-segment resolution; T1D—type 1 diabetes mellitus; T2D—type 2 diabetes mellitus; TG—triglycerides; TLR-2,4—toll-like receptor 2,4; TRAP—thrombin receptor-activating peptide; TXA2—thromboxane A2; UA—unstable angina; VTE—venous thromboembolism. ^1^ Active metabolite.
