# Correction: Pluta et al. Platelet–Leucocyte Aggregates as Novel Biomarkers in Cardiovascular Diseases. *Biology* 2022, *11*, 224

**DOI:** 10.3390/biology11111554

**Published:** 2022-10-24

**Authors:** Kinga Pluta, Kinga Porębska, Tomasz Urbanowicz, Aleksandra Gąsecka, Anna Olasińska-Wiśniewska, Radosław Targoński, Aleksandra Krasińska, Krzysztof J. Filipiak, Marek Jemielity, Zbigniew Krasiński

**Affiliations:** 1 1st Chair and Department of Cardiology, Medical University of Warsaw, Banacha 1a, 02-097 Warsaw, Poland; 2 Department of Cardiac Surgery and Transplantology, Poznan University of Medical Sciences, 61-701 Poznan, Poland; 3 1st Department of Cardiology, Medical University of Gdansk, 80-210 Gdansk, Poland; 4 Department of Ophtalmology, Poznan University of Medical Sciences, 61-701 Poznan, Poland; 5 Department of Clinical Sciences, Maria Sklodowska-Curie Medical Academy in Warsaw, 00-136 Warsaw, Poland; 6 Department of Vascular and Endovascular Surgery, Angiology and Phlebology, Poznan University of Medical Sciences, 61-701 Poznan, Poland

The authors would like to make the following correction to the published paper [1].

## Error in Figure

In the original publication, there was a mistake in Figure 1 as published. There was an accidental exchange in the caption of P-selectin (which should be on platelet surface) and PSGL-1 (which should be on leucocyte) during the editing of Figure 1. The corrected Figure 1 appears below.

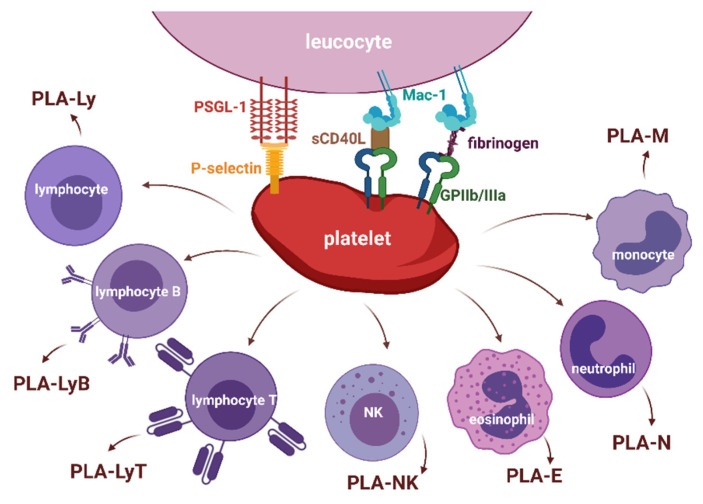



The authors apologize for any inconvenience caused and express their sincere appreciation to the scholars who pointed out the mistake. This correction was approved by the Academic Editor. The original publication has also been updated.
